# The Ability of Zika virus Intravenous Immunoglobulin to Protect From or Enhance Zika Virus Disease

**DOI:** 10.3389/fimmu.2021.717425

**Published:** 2021-09-06

**Authors:** Amelia K. Pinto, Mariah Hassert, Xiaobing Han, Douglas Barker, Trevor Carnelley, Emilie Branche, Tara L. Steffen, E. Taylor Stone, Elizabeth Geerling, Karla M. Viramontes, Cory Nykiforuk, Derek Toth, Sujan Shresta, Shantha Kodihalli, James D. Brien

**Affiliations:** ^1^Department of Molecular Microbiology and Immunology, Saint Louis University, St Louis, MO, United States; ^2^Emergent BioSolutions Canada Inc, Winnipeg, Canada; ^3^La Jolla Institute for Immunology, Center for Infectious Disease and Vaccine Research, La Jolla, CA, United States

**Keywords:** Zika virus, dengue virus, hyperimmunoglobulin, antibody dependent enhancement, animal model, antibody dependent enhancement

## Abstract

The closely related flaviviruses, dengue and Zika, cause significant human disease throughout the world. While cross-reactive antibodies have been demonstrated to have the capacity to potentiate disease or mediate protection during flavivirus infection, the mechanisms responsible for this dichotomy are still poorly understood. To understand how the human polyclonal antibody response can protect against, and potentiate the disease in the context of dengue and Zika virus infection we used intravenous hyperimmunoglobulin (IVIG) preparations in a mouse model of the disease. Three IVIGs (ZIKV-IG, Control-Ig and Gamunex^®^) were evaluated for their ability to neutralize and/or enhance Zika, dengue 2 and 3 viruses *in vitro*. The balance between virus neutralization and enhancement provided by the *in vitro* neutralization data was used to predict the IVIG concentrations which could protect or enhance Zika, and dengue 2 disease *in vivo*. Using this approach, we were able to define the unique *in vivo* dynamics of complex polyclonal antibodies, allowing for both enhancement and protection from flavivirus infection. Our results provide a novel understanding of how polyclonal antibodies interact with viruses with implications for the use of polyclonal antibody therapeutics and the development and evaluation of the next generation flavivirus vaccines.

## Introduction

Zika virus (ZIKV) is an arbovirus that belongs to the genus *Flavivirus*, which includes important human pathogens including West Nile virus (WNV), and the four serotypes of dengue virus (DENV1-4) (1). ZIKV was first isolated from a macaque in the Zika forest of Uganda in 1947 (2); following its isolation and identification, there have been periodic reports of human ZIKV infections in Africa and Asia (3–13). From 2007-2013 there were substantial outbreaks of ZIKV infections in regions that had not previously had evidence of ZIKV circulation (14–16). In 2015 the World Health Organization (WHO) reported outbreaks of ZIKV in the Americas (17). Beginning with an outbreak in 2013, our understanding of the risk and severity of ZIKV disease changed (17). Studies have suggested that the increase in ZIKV virulence is associated introduction of the virus into a naïve population combined with the ability of the virus to persist for extended periods in body fluids expanding the window for ZIKV to be sexually transmitted (18–20). Sexual transmission and mosquito associated infection increases the risk of fetal ZIKV infection leading to congenital Zika virus syndrome (CZS) (21–23). There continues to be active Zika virus (ZIKV) transmission and currently there are no FDA-approved treatments or vaccines to prevent. ZIKV infection or disease. The protracted ZIKV transmission window and the expanding list of ZIKV associated neurological sequelae has driven our efforts to develop Anti-Zika Immune Globulin (Human) (ZIKV-IG), a passive immunoglobulin therapy to prevent ZIKV associated diseases.

Hyperimmune immunoglobulins are highly purified concentrated IgG preparations generated from a pool of individual plasma donors selected due to antibody specificity and function for the use as a therapeutic. Passive immunoglobulin therapy is a well-established, and effective method for the treatment of disease [Reviewed in (24)]. The protective capacity of passively transferred pathogen specific immunoglobins was shown almost 150 years ago, and the passive administration of immunoglobulins for the protection of immune deficient populations began in the 1950s [Reviewed in (25)]. Clinical use of passive immunoglobulin therapies has also been established to prevent disease including birth defects caused by other viruses such as rubella virus, varicella-zoster virus, or cytomegaloviruses (26–28), and are currently under investigation for multiple Beta coronaviruses including SARS-CoV-2 infection (29, 30). WNV hyperimmunoglobulin has also been shown to be effective in reducing disease severity and improving outcomes for patients with WNV associated encephalitis (31).

As mentioned above, WNV, and ZIKV are both flaviviruses, and with the previous studies noting an efficacy of hyperimmunoglobulin for improving disease outcomes with WNV infections, it would seem highly likely that a similar result could be expected for ZIKV. However, the enthusiasm for hyperimmunoglobulin treatment of flavivirus infection is dampened by the well-documented history of increased disease severity seen among the dengue viruses associated with existing antibodies to DENV 1-4. The closely related flaviviruses, DENV1-4 are four serologically distinct, mosquito-borne viruses that infect an estimated 400 million people each year, according to the WHO. While primary DENV infection is believed to confer long-term immunity against homologous virus infection, human epidemiological data indicates that people who have a secondary infection with a heterologous DENV serotype have an increased risk of severe DENV disease development [(32, 33), reviewed in (34)]. One of the main drivers of this increased risk of disease is thought to be antibody-dependent enhancement (ADE), where non- or poorly-neutralizing antibodies generated during primary infection fail to neutralize the secondary serologically distinct virus, and instead, cause enhanced infectivity of target cells through virus opsonization and Fc-receptor-mediated endocytosis (35–37). Highlighting the potential risk of ADE by the administration of antibody, previous studies with both non-human primates and mice have shown that administration of low concentration or poorly neutralizing DENV specific antibodies prior to infection with DENV can cause ADE in these animal models (38–42).

ZIKV shares an approximate 60% sequence identity with DENV1-4 (43), co-circulates in the same geographic area and infection with either ZIKV or DENV1-4 can generate cross-reactive antibody responses to the other (44–48). Since the expansion of the geographic distribution of ZIKV starting in 2007, the immunological interactions that can lead to ADE have increased because of the close structural similarities between DENV1-4 and ZIKV. For the therapeutic development of passive immunoglobulin therapies, it is important to understand whether ZIKV-IG has any role in modulating subsequent ZIKV or DENV infections in subjects exposed to either of these infections after treatment with ZIKV-IG. *In vitro* studies have shown that dengue antibodies can potentiate ZIKV infection and inversely that ZIKV antibodies can potentiate dengue infection (49–51); similar studies *in vivo* using small animal models and nonhuman primates have achieved conflicting results, with some studies demonstrating protection [(52), reviewed in (53)] and other studies showing increased disease (54–56).

To define the boundary of therapeutic efficacy and potential for ADE, we investigated the ability of ZIKV-IG to neutralize or enhance ZIKV and DENV infection *in vitro* and *in vivo*. We determined that the ZIKV-IG contained both cross-reactive neutralizing and enhancing antibodies. By overlaying the *in vitro* ZIKV-IG neutralizing and enhancing curves, we determined where ADE could be dominant over the potential for neutralization, thereby defining a window of caution. At a dose within this window of caution, we demonstrated that ZIKV-IG was able to enhance ZIKV disease in a mouse model. To understand the mechanism of ZIKV-IG enhancement, we evaluated clinical hematology and viral titer at multiple time points post-infection, showing that the ADE we observed led to increases in viral load. We noted that the human polyclonal antibody response is capable of driving enhanced ZIKV disease and that disease potential occurs when the concentration of polyclonal antibody is at the concentration of peak enhancement *in vitro*.

## Materials and Methods

### Ethics Statement

This study was carried out in accordance with the recommendations in the Guide for the Care and Use of Laboratory Animals of the National Institutes of Health. The protocols were approved by the Institutional Animal Care and Use Committee at the Saint Louis University (Assurance Number: D16-00141).

### Viruses and Cells

The ZIKV strain, PRVABC59, used for these experiments was a kind gift of Robert Lanciotti (CDC). Asian lineage strain, FSS13025, was isolated in 2010 from a pediatric case (57). It was obtained from the World Reference Center for Emerging Viruses and Arboviruses (WRCEVA). The DENV-2 strain (D2S20) is a mouse-adapted strain and has been described previously (41). The DENV-3 strain (Co360/94) is a non-mouse-adapted DENV-3 Thai human isolate (58). All viruses used were grown in Vero cells and ultracentrifuged [30,000 x g, 3 hours, SW32 rotor (110,500 x g)] through a 25% glycerol cushion. Pelleted virus was resuspended in 10 mM Tris pH 8.0, 150 mM NaCl, and 1 mM EDTA, and stored at -80°C. Virus was diluted in PBS prior to infection. Virus stocks were titered by a focus-forming assay on Vero cells (39). ADE assays were completed on K562 cells, a human leukemia cell line (Kind gift of Dr. Jacki Kornbluth, Saint Louis University).

### Polyclonal and Monoclonal Antibody Therapeutics

Three IVIG products were utilized in this study, ZIKV-IG, Control-Ig, and Gamunex^®^. ZIKV-IG and Control-Ig were provided by Emergent BioSolutions at a concentration of 93 mg/mL and 53 mg/mL of purified IgG, respectively. ZIKV-IG was purified IgG derived from human plasma collected at US FDA licensed plasma centers and were identified as Zika virus antibody positive by antibody neutralization assay as described previously for DENV and ZIKV (52, 59, 60). The Control-Ig was generated at Emergent BioSolutions using the same process as the ZIKV-IG product. Gamunex^®^, an FDA approved product used as a treatment for primary humoral immunodeficiency, was at a concentration of 100 mg/mL. Two monoclonal antibodies products were used, 4G2 mAb a pan flavivirus-reactive antibody, and an IgG2a mAb Isotype Control (BioXCell^®^).

### Antibody Neutralization Assay

Focus forming reduction neutralization assays (FRNT) were performed in a similar manner to those described (61) and were used to determine the 50% and 90% effective neutralizing concentration of immunoglobulins against Zika virus and DENV. Briefly, four-fold serial dilutions of antibody (2.5 mg/mL to 19 ng/mL) were mixed with ~80 focus forming units (FFU) of virus, incubated at 37°C for 1 hour, then added to Vero cell monolayers in 96 well plates for 1 hour at 37°C to allow virus adsorption. Cells were overlaid with 1% methylcellulose and incubated for two days. Monolayers were fixed for 1 hour at room temperature, washed and permeabilized. Infected cell foci were stained by incubating cells with 500 ng/mL of flavivirus cross-reactive mAb 4G2 for 1 hour at 4°C, washed and detected by incubating cells with a 1:5000 dilution of horseradish peroxidase conjugated goat anti-mouse IgG for 1 hour. After washing, staining was visualized by addition of TrueBlue™ detection reagent. Infected foci were enumerated using a CTL-Immunospot S6 (Cellular Technology Limited).

### Human Monocyte Isolation and Differentiation in Human Monocyte-Derived Macrophage

Human peripheral blood mononuclear cells were separated from whole blood obtained by venipuncture from healthy volunteers deidentified under the La Jolla Institute Internal Review Board Protocol VD-057-0217. Donors were HIV, hepatitis B, and hepatitis C negative. Human peripheral blood mononuclear cells were obtained after density centrifugation (400 × g for 30–60 min at 4°C) using Histopaque 1077 (Sigma). Monocytes were then negatively selected from the buffy coat using the pan monocyte isolation kit, human (Miltenyi Biotech) as described by the manufacturer. Cells were plated into 24 well plates (4.0 x 10^5^ cells/well) and differentiated for 7 days in complete macrophage medium [macrophage serum-free medium (Gibco) supplemented with 1% penicillin/streptomycin, 1% Nutridoma™-SP (Roche), 1% fungizone (Gibco), and 100 ng/mL human macrophage-colony stimulating factor (M-CSF) (PeproTech)] at 37°C, 5% CO_2_. The medium was changed every 2–3 days.

### Antibody Dependent Enhancement Assay

ADE assays were performed in a similar manner for both K562 and human derived monocytes, cell types for each assay are noted within the figure legends. The K562 ADE assays were analyzed using procedures described in Pantoja et al. (2017) to quantify the antibody-mediated enhancement potential of human polyclonal antibody immunoglobulin specific for ZIKV (ZIKV-IG) in comparison to commercially available immunoglobulin, (Gamunex^®^ and Control-Ig) and a pan-flavivirus monoclonal antibody (4G2) known to mediate ADE *in vitro* and *in vivo* (38, 56). Four-fold serial dilutions of antibody (2.5 mg/mL to 2.4 ng/mL) were mixed with ZIKV or DENV at a multiplicity of infection (MOI) of 1 then incubated at 37°C for 1 h, to allow the formation of immune complexes. Immune complexes were added to K562 cells that express the Fc-gamma receptor (FcγR) CD32A and incubated for 2–3 days. Cells were fixed, washed, permeabilized, and stained with flavivirus cross-reactive mAb 4G2. Cells were stained with A647-conjugated goat anti-mouse IgG secondary antibody, washed, and analyzed on an Attune flow cytometer. The percentage of infected cells was determined using FlowJo 10.4.2 software (Tree Star, Ashland, OR). ADE was calculated as [% infected cells in the presence of antibody or serum]/[% infected cells in of virus only (no antibody or serum)].

For ADE assays using HMDM cells, ZIKV strain FSS13025 was incubated with ZIKV-IG or Gamunex^®^ as described in the figure (**Figure S2**) then serially diluted 1:5 for 5 dilutions in macrophages medium. As a positive control DENV human immune serum [0.6% (vol/vol)] was incubated with virus. The virus/antibody or virus/serum mixtures were incubated with HMDMs for 2 h at 37°C, 5% CO_2_. The supernatant was removed and fresh warm complete macrophage medium was added. After 22 h incubation, HMDMs were resuspended, fixed, stained and analyzed in a manner similar to the K562 ADE assay.

### Animal Infections and Treatments

#### ADE Studies

*Ifnar1-/-* mice homozygous for interferon alpha and beta receptor subunit knockout mutation on the C57BL/6J background (B6.129S2-Ifnar1^tm1Agt^/Mm) were from the Jackson Laboratory. Mice were bred in a pathogen-free mouse facility at the Saint Louis University School of Medicine and experiments were performed in accordance with and approval of Federal and University regulations. To induce ADE, mice were treated with the flavivirus cross-reactive antibody 4G2 (62) at 20 μg/mouse, Gamunex^®^ at 2 mg/mouse, and ZIKV-IG at 50 μg/mouse *via* intraperitoneal route. 24 ± 4 hours after antibody treatment, ZIKV and DENV2 were diluted in sterile PBS to obtain a final concentration of 1 x 10^4^ FFU of DENV-2 (D2S20) or 1 x 10^5^ FFU of ZIKV (PRVABC59) per mouse. For the *in vivo* DENV-2 studies, 4 week old mice were challenged by intravenous (IV) injection of 100 μL of virus, which was a dose and route optimized within our lab to allow viral replication and measurable disease, but not mortality (38). For *in vivo* ZIKV studies, 8 week old mice were challenged by subcutaneous (SC) footpad injection of 50 μL of virus, which was dose and route optimized within our lab to allow viral replication and measurable disease, but not mortality (61, 63). Mice were anesthetized during this procedure using Ketamine/Xylazine (90 mg/kg: 10 mg/kg). After challenge, each mouse was examined for visible trauma and placed back into its cage for recovery.

### Clinical Monitoring

Animals were observed for clinical outcomes including body weight, activity and mortality daily after receiving antibody. Animal activity assessments from 0-6 were recorded for each animal as follows: Paralysis Scoring: 0=Clinically normal; 1=Tail tone, flaccid tail; 2=Hind limb weakness, grasping, and balance; 3=Hind limb paraplegia, inability to move hind limbs or urinary incontinence; 4=Weakness of fore limbs, in addition to hind limb dysfunction and/or distended abdomen; 5=Quadriplegia, inability to move hind limbs and forelimbs; 6=Moribund, animal is not moving.

#### Measurement of Viral Burden

On days 4 and 8 post-infection, intracardiac perfusion (20 mL of PBS) was performed and organs were recovered. Blood was collected in EDTA coated tubes. Organs were harvested in 1.5 mL Eppendorf tubes and snap frozen, weighed, and homogenized in DMEM using a BeadMill 24 from Fisher Scientific. Measurement of infectious virus was completed by focus forming assay, similar to the focus reduction neutralization assay, and described previously (59).

### Clinical Hematology and Chemistry Analysis

On days 4 and 8 post-infection, mice were terminally bled into EDTA tubes and hematological parameters were assessed. Blood for clinical hematology was collected into EDTA coated tubes (Beckton Dickinson) and analyzed using a ProCyte DX machine (IDEXX).

### Statistical Analysis Methods

#### Survival

Time to death was defined as the number of days from the virus challenge on day 0 to the date of death. Analysis of mean time-to-death, median time-to-death for each treatment group versus 4G2 were tabulated using Kaplan-Meier estimates of mean time-to-death, median time-to-death and Dunnett’s test for survival time distributions for each treatment group versus 4G2.

#### Weight Change

Mean weight change from baseline to study day 5 were compared for all experimental groups using a nonparametric Kruskal-Wallis test and adjusted for multiple comparisons. Primary comparison was each treatment group to 4G2 control with the isotype control being the secondary comparator.

#### Paralysis Scoring

Analysis of clinical scores was a secondary endpoint and analysis of groups was compared on day 5 post infection. A non-parametric Kruskal-Wallis test was used to test for a significant difference with the primary comparison being each treatment group to 4G2 control with the isotype control being the secondary comparator. Analysis of rates of animals in each treatment group with a score of 1 or higher at any time on study was also completed for this analysis the Fisher’s exact test was used and all groups were compared to 4G2. Statistical significance has been indicated within the figures with asterisks (*p< 0.05, **p<0.005, ***p<0.0005, ****p<0.0001).

## Results

While it has been shown that ZIKV-IG is protective at doses equal to and greater than 10 mg/kg in ZIKV infected IFNAR1-/- mice (52), studies with DENV (38, 40) suggest that at non-sterilizing concentrations, a polyclonal hyperimmune serum such as ZIKV-IG could induce ADE. The primary objective of this study was to evaluate the effect of ZIKV-IG at a sub-neutralizing concentration on subsequent flavivirus infection to determine the likelihood that the ZIKV-IG would induce ADE both *in vitro* and *in vivo* and to understand the quantitative relationship between the *in vitro* and *in vivo* results, defining a window of caution.

### Intravenous Hyperimmunoglobulin Neutralization of Flaviviruses *In Vitro*

**Figure 1:**
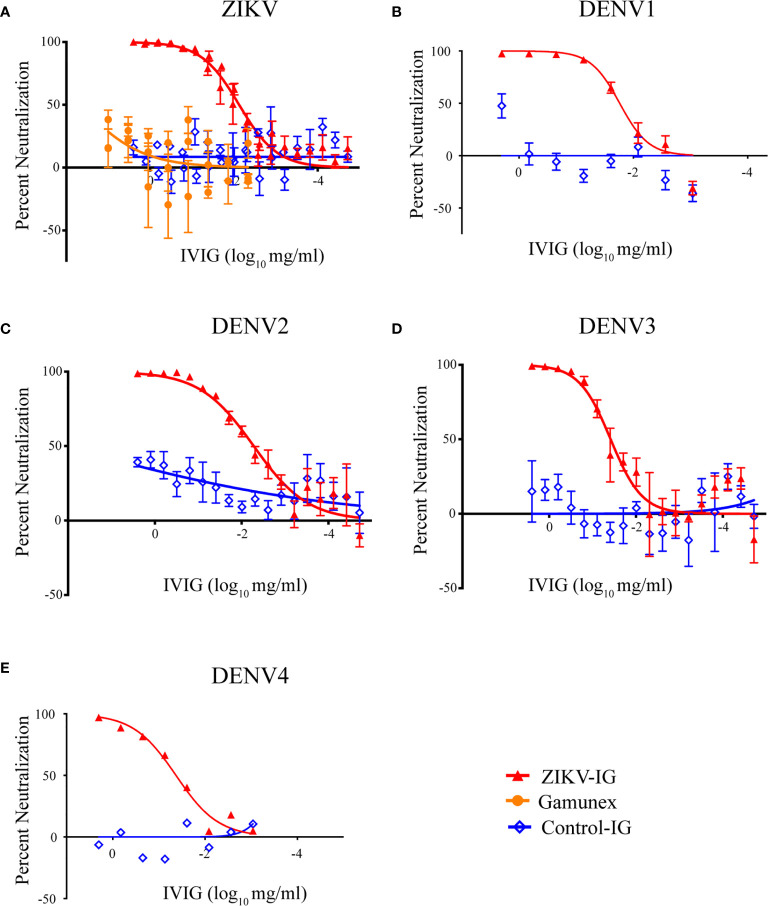
Human IVIG can neutralize ZIKV and DENV1-4. The ability of IVIG products, ZIKV-IG, Control-Ig, and Gamunex^®^ to neutralize **(A)** Zika virus strain PRVABC59, **(B)** DENV1, **(C)** DENV2, **(D)** DENV3 and **(E)** DENV4 were completed by focus reduction neutralization test using Vero cells. Serial dilutions of serum were incubated with a constant amount of virus and individual foci were counted. Foci counts are normalized to the number of foci in PBS control wells. Data shown as single points that represent the mean of 3-4 independent experiments completed in duplicate +/- SEM. Solid lines display the nonlinear regression log vs. response with variable slope.

A Vero-based focus reduction neutralization test (FRNT) was used to define the neutralizing and sub-neutralizing concentrations of ZIKV-IG *in vitro* (**Figure 1A**). For controls, we used two additional hyperimmunoglobulin products: a non-specific-Ig manufactured by Emergent BioSolutions Canada Inc. as a production control referred to as Control-Ig, and Gamunex^®^, an FDA approved widely used IVIG with a primary indication for primary humoral immunodeficiency and chronic inflammatory demyelinating polyneuropathy and was used as a biosimilar control. By FRNT, low levels of ZIKV neutralization were observed with Gamunex^®^ when used at a range of ~10-1 mg/mL, a concentration range that resulted in a peak neutralization of 20%, while no ZIKV neutralization was observed with Control-Ig at the same range of concentrations. The neutralization potential of ZIKV-IG had an effective concentration 50 (EC_50_) of 0.007 mg/mL and an EC_90_ of 0.074 mg/mL (**Table 1**). Supporting the previously reported results by Branche et al. (52), ZIKV-IG neutralized virus completely at concentrations that ranged from 2.5 mg/mL to 0.3 mg/mL. The level and concentration range is similar to hyperimmune Vaccinia Immune Globulin (64, 65), a result supporting ZIKV-IG viability as a potential therapeutic.

**Table 1:** Analysis of neutralization effective concentration of ZIKV-IG against ZIKV and the four serotypes of DENV.

	LogEC_50_ mg/mL	SEM	Log EC_90_ mg/mL	SEM
ZIKV	-2.13	0.032	-1.13	0.098
DENV1	-1.76	0.061	-1.20	0.13
DENV2	-2.28	0.084	-0.92	0.184
DENV3	-1.42	0.088	-0.62	0.192
DENV4	-1.37	0.081	-0.36	0.178

To determine the *in vitro* neutralization potential of ZIKV-IG and Control-Ig on DENV serotypes 1-4, we again assessed the neutralization potential in Vero cells by FRNT. The ZIKV-IG EC_50s_ for the four DENV serotypes were between 0.005 and 0.042 mg/mL with a hierarchy of neutralization that showed ZIKV=DENV2>DENV1>DENV3>DENV4 (**Figures 1B-E** and **Table 1**). Similar to what we had previously observed for ZIKV, no neutralization was observed with Control-Ig at the same range of concentrations. For all serotypes of DENV, the ZIKV-IG is capable of neutralizing 100% of the virus population indicating that the antibody repertoire contained within the ZIKV-IG has a breadth of specificities or concentrations to neutralize a range of flaviviruses.

### Enhancement of Flaviviruses *In Vitro*

**Figure 2:**
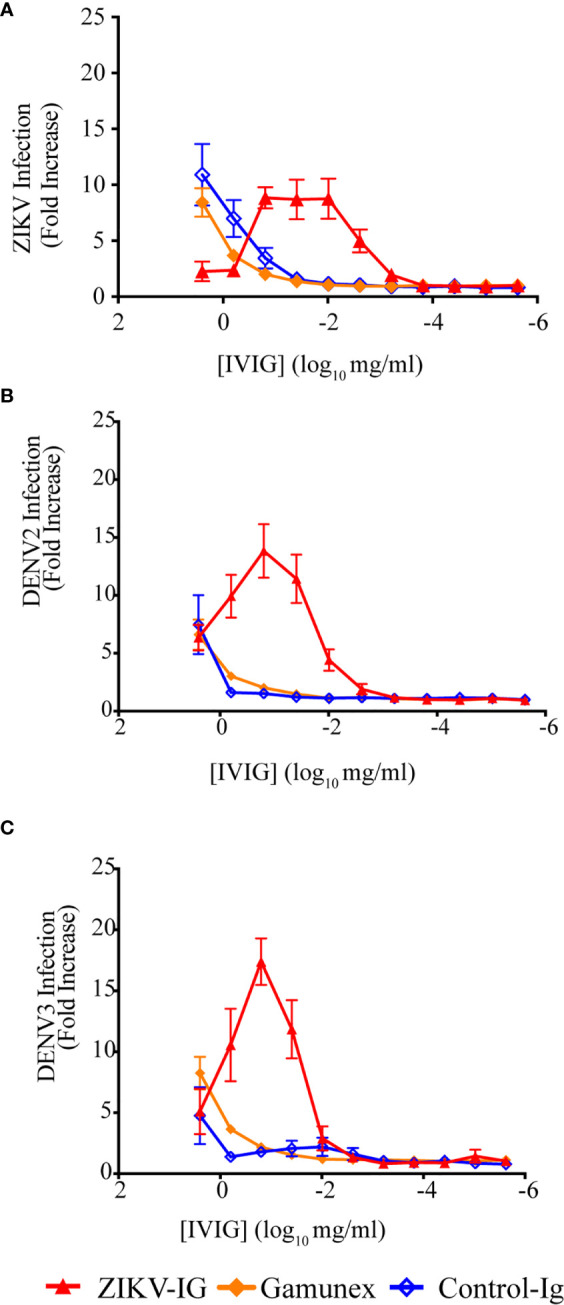
ZIKV-IG *in vitro* enhancement of ZIKV and DENV serotypes 2 and 3. The ability of three IVIG products, ZIKV-IG, Control-IG, and Gamunex^®^ to enhance **(A)** ZIKV, **(B)** DENV2 and **(C)** DENV3 infection in K562 cells. For antibody enhancement serial dilutions of IVIG were incubated with a constant amount of virus then added to K562 Fc expressing cells. Then infected cells were enumerated by flow cytometry. The base line infection of K562 cells was 2.43% +/- 1.44 for ZIKV, 3.78% ± 2.26% for DENV2 and 2.12% ± 1.09% for DENV3. Fold enhancement data is shown as single points that represent the mean of 3-4 independent experiments completed in duplicate +/- SEM.

With the *in vitro* neutralization concentrations for ZIKV and all four serotypes of DENV established, we next set out to determine the ability of the ZIKV-IG to enhance flavivirus infection (**Figure 2** and **Table 2**). To measure enhancement potential, we utilized a K562 ADE assay that has been used by our group and others to assess the Fc mediated uptake of virus, leading to an increased infection of cells (56). In these studies, we assessed the potential of ZIKV-IG, Gamunex^®^, and Control-Ig to enhance ZIKV (**Figure 2A**), DENV2 (**Figure 2B**) and DENV3 infections (**Figure 2C**). As a control, we performed similar experiments using the mAb 4G2, which is known to enhance DENV infection *in vitro* (66) (**Figure S1**) as well as DENV infection and disease *in vivo* (40) + 38.

**Table 2:** Analysis of antibody dependent enhancement of ZIKV-IG against ZIKV and DENV2 and 3.

	Max fold Enhancement	SEM	Mean IVIG concentration (mg/mL)
ZIKV	9.59	2.98	0.03
DENV2	13.58	2.31	0.159
DENV3	17.47	1.91	0.156

The studies evaluating the potential of ZIKV-IG to enhance ZIKV replication *in vitro* demonstrated a dose-dependent increase in ZIKV infection with an enhancement of 9.59+/-2.98 fold (mean +/- SEM) over background between the concentration of 0.156 mg/mL and 0.097 mg/mL (**Figure 2A** and **Table 2**). We also observed a dose-dependent effect in ZIKV replication enhancement with Control-Ig at higher concentrations with a 4.27+/-1.29 fold enhancement at 0.63 mg/mL and 9.57+/- 3.14-fold enhancement at 2.5 mg/mL. At similarly high concentrations, we noted that the Gamunex^®^ had a 3.36+/- 1.83-fold enhancement at 0.63 mg/mL and 7.23+/-1.58-fold enhancement at 2.5 mg/mL. Based upon the low level of neutralization and enhancement of ZIKV and DENV by Gamunex^®^ and the previous finding of anti-flavivirus antibodies within IVIG preparations (67), we tested Gamunex^®^ for anti-DENV IgG (InBios) by ELISA at a 1:20 dilution and obtained a positive result, demonstrating that the cause of this observed neutralization and enhancement was likely the presence of flavivirus reactive antibodies in those IVIG preparations (data not shown).

These results showed that polyclonal ZIKV-IG was able to enhance ZIKV infection over approximately a two log range of concentrations in the K562 enhancement assay. However, we next wanted to understand the range of concentrations ZIKV-IG could enhance ZIKV infection in primary human monocyte derived macrophages (HMDMs). We generated HMDMs from six separate donors as targets in the ADE assay (**Figure S2**). ZIKV-IG enhanced ZIKV infection of HMDMs from 13-27-fold above background compared to placebo at 0.008 and 0.0016 mg/mL; while no enhancement was observed at any concentration with Gamunex^®^. These data illustrate that *in vitro* dependent enhancement of ZIKV can occur in primary cells and over a similar range of antibody concentrations that occur with K562 cells.

Our results from the *in vitro* enhancement assays demonstrate the ability of the ZIKV-IG to enhance ZIKV, DENV2, and DENV3 infection over a two-log range of concentrations. This breadth of concentrations driving *in vitro* ADE has been observed with human serum previously (54, 68), and may speak to the range of structural conformations of the virion or antibody specificities. Based on previous studies, ZIKV and DENV contain a range of structural conformations that affect the antigen/epitope availability [reviewed in (69)] from immature virions containing prM and mature virions with increased exposure of quaternary epitopes (70–72).

### Calculation of a Dose of ZIKV-IG Likely To Induce ADE in *Ifnar1-/-* Mice

**Figure 3:**
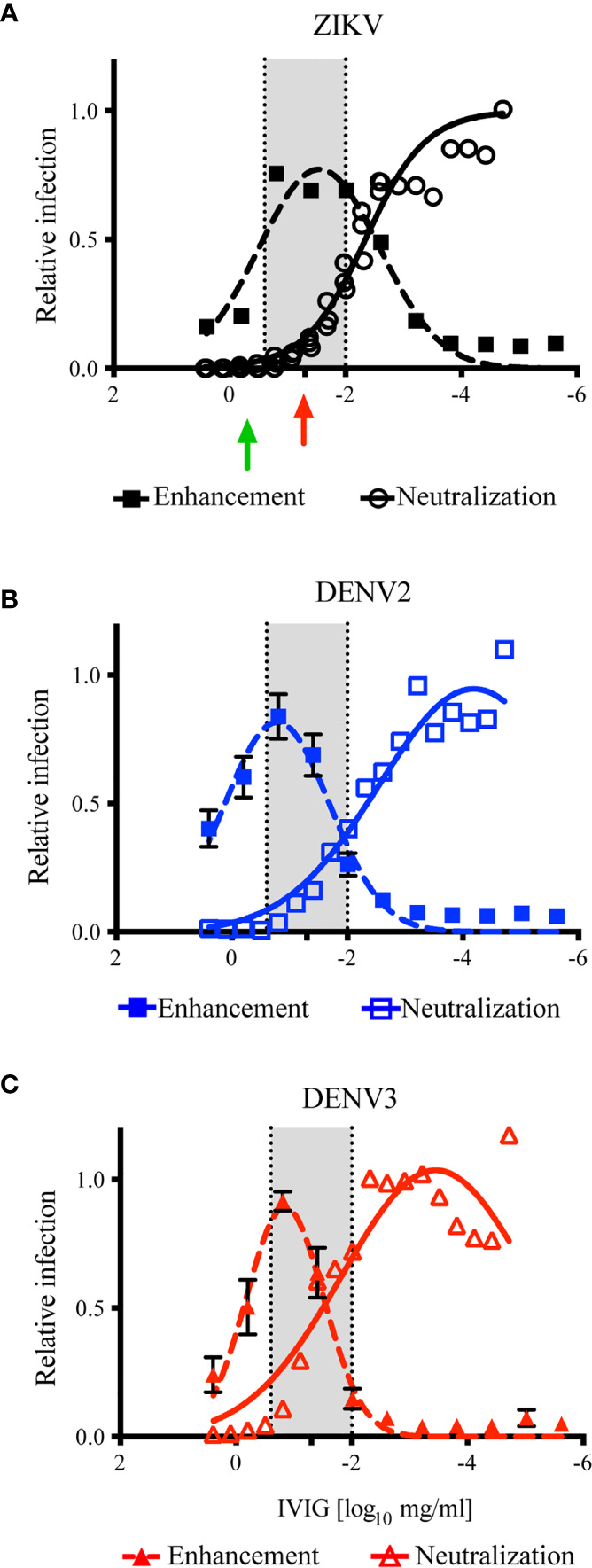
Calculation of ZIKV-IG disease enhancement dose. The overlay of the results of ZIKV-IG antibody neutralization in Vero cells (open symbols) and antibody enhancement in K562 cells (closed symbols) for ZIKV, DENV2 and DENV3. The *in vitro* neutralization data from **Figure 1** was normalized to 1 = 100% infection. The *in vitro* ADE data in **Figure 2** was normalized to the peak of infection for each respective virus, with 9.59 fold enhancement for ZIKV at a peak at 0.03 mg/ml, 13.58 fold enhancement for DENV2 at 0.159mg/ml, and 17.47 fold enhancement for DENV3 at 0.156mg/ml. The ADE data was then fit using a gaussian dose response function, to identify the peak of ADE activity and to define where the ADE concentration crossed antibody neutralization. A region where antibody neutralization begins to decrease and antibody enhancement increases is highlighted in grey, to outline the potential dose curve where subjects could be at risk (249mg/ml to 0.01mg/ml). **(A)** The antibody neutralization and ADE of ZIKV by the ZIKV-IG is presented. The green arrow is the concentration of antibody that has been shown by Bardina et al. to protect against ZIKV disease and the red arrow represents the dose chosen to evaluate for the potential enhancement of disease *in vivo*. **(B)** The antibody neutralization and ADE of DENV2 by the ZIKV-IG is shown. **(C)** The antibody neutralization and ADE of DENV3 by the ZIKV-IG is shown.

Previous structural and functional studies of mAbs suggest that any individual antibody has the potential to cause ADE *in vitro* [reviewed in (73)]. Using *in vitro* systems, we are able to experimentally determine the IgG concentration of complex polyclonal sera or hyperimmunoglobulin that can cause ADE *in vitro*; yet it is difficult to determine the concentration of polyclonal Ig which may cause ADE *in vivo* [reviewed in (74)]. To predict the *in vivo* treatment dose that could have the greatest potential for ZIKV ADE, the antibody neutralization results (**Figure 1**) were overlaid with the *in vitro* ADE results (**Figure 2**). To focus on the concentration of ZIKV-IG that was either neutralizing or enhancing, we normalized the ADE curve to the maximum enhancement for the assay and overlaid the two results. Upon overlaying the ZIKV antibody neutralization and ADE results (**Figure 3A**), the peak of ADE activity was observed at the IVIG concentration in which ~83% of the virus was neutralized (**Figure 1**). This result was surprising because based upon previous studies completed with mAbs (75), one would predict that ADE would occur at ~EC_50_ of the neutralizing antibody response. This observation suggests the enhancement and neutralization behavior of complex polyclonal sera is fundamentally different than that of mAbs. In order to investigate this further, we overlaid the antibody neutralization and ADE results for DENV2 (**Figure 3B**) and DENV3 (**Figure 3C**) where the peak of the ADE activity was observed when ~97% and ~ 92% of the virus was neutralized.

Based upon the evaluation of the overlapping regions, we calculated a range of 0.25 to 0.010 mg (highlighted in grey in **Figure 3**) would be predicted to cause ADE with ZIKV *in vivo* and increase disease severity in our animal model (61). As the *in vitro* neutralization and ADE activity of ZIKV-IG for DENV2, DENV3, and ZIKV were similar, we predicted we would observe ADE *in vivo* in our animal model for DENV2 (38). A dose of 0.050 mg/mouse (~3.85 mg/kg) of ZIKV-IG fell within the range that was predicted to drive ADE disease for ZIKV, DENV2, and DENV3 (**Table 3**). As a positive control for DENV *in vivo* ADE, we utilized a dose of 20 µg/mouse of 4G2, a dose our group and others have shown to cause ADE *in vivo* (38, 40, 76).

**Table 3:** Analysis of viral titer in ZIKV-IG treated mice 3 and 7 days post Zika virus infection.

Tissue	Treatment Group	Day 3			Day 7		
		Male	Female	Male + Female	Male	Female	Male + Female
Kidney	ZIKV-IG 0.50 mg/mouse	0.034*	0.403	0.092	0.424	1.000	1.000
	ZIKV-IG 0.05 mg/mouse	0.037*	0.095	0.022*	0.424	1.000	1.000
	ZIKV-IG 0.01 mg/mouse	0.095	0.060	0.030*	0.424	1.000	1.000
Serum	ZIKV-IG 0.50 mg/mouse	0.456	0.011*	0.106	1.000	0.180	0.703
	ZIKV-IG 0.05 mg/mouse	0.012*	0.205	0.004*	0.441	0.424	0.703
	ZIKV-IG 0.01 mg/mouse	0.141	0.672	0.715	0.044*	0.072	0.015*

*Significant Wilcoxon rank sum p-value (<= 0.05).

### Enhancement Potential of the ZIKV-IG *In Vivo* in *Ifnar1-/-* Mice Infected With ZIKV

To evaluate the potential risk of ADE resulting from ZIKV-IG administration, eighty *Ifnar1-/-* mice (8 to 10 weeks old, 40 males and 40 females) were divided into four different treatment groups (10 males and 10 females per group) and treated with ZIKV-IG, Gamunex^®^ (hyperimmune control), 4G2 (positive control) or Isotype mAb (negative control) 24 ± 4 hours prior to infection (**Table 3**). 4G2 can induce ADE with DENV *in vitro* and *in vivo* (38, 40, 76–81), although our prior unpublished experiments had suggested that 4G2 was not likely to cause ADE if administered prior to ZIKV infection. Twenty-four hours after IVIG or mAb treatment, the *Ifnar1-/-* mice were challenged *via* subcutaneous route (SC) with 1 x 10^5^ FFU/mouse of ZIKV strain PRVABC59. The data from our previous studies in *Ifnar1-/-* mice demonstrated that in the absence of treatment or intervention, at this dose of ZIKV we would observe mild signs of disease including body weight loss and mild clinical disease, however, we would observe low or no (0 to 15%) mortality (61, 63).

**Figure 4:**
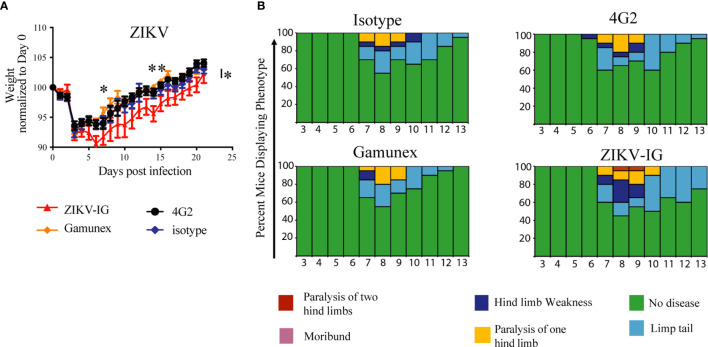
Weight loss, and clinical score of ZIKV-infected *Ifnar1-/-* mice under ADE conditions. *Ifnar* ^-/-^ mice were transferred passively isotype control (0.020 mg of mouse anti-NP or 2 mg of Gamunex^®^) or enhancing mAbs (0.020 mg of anti-E (4G2) or 0.050 mg of ZIKV-IG) and then infected a day later with 10^5^ FFU of ZIKV (strain PRVABC59) *via* an intravenous route. Weight change **(A)** and clinical score **(B)** were monitored. The data reflects 20 mice per group, consisting of 10 males and 10 females. Mice were evaluated for signs of disease daily and graphed on each day as a percentage of mice displaying that disease indicator. Signs of disease range from no apparent disease, limp tail, hind limb weakness, hind limb paralysis, complete paralysis to death. Body weight comparison as single days was completed by Dunnett’s test, comparisons of weight over time was completed by 2-way ANOVA, while number of disease days was determined by Fisher exact test. Statistical differences are denoted by a star *p < 0.05, **p < 0.005.

Following ZIKV infection the mice were monitored daily for 21 days for mortality, weight loss, and clinical signs of disease (**Figure 4**). To reduce bias into these studies, the antibody and IVIG treatments were prepared and administrated by an independent investigator who did not participate in clinical monitoring for the study. For the clinical scoring, a numeric score, described more thoroughly in the methods section, was assigned daily to each mouse from day 0 through day 21 based on indicators of disease that we have previously published in this model including limb weakness and paralysis (61, 63). The greatest decrease in body weight was seen for all treatment groups between days two and three post-infection. The loss of body weight between days two and three was anticipated, as it indicates establishment of infection of the susceptible *Ifnar1-/-* mice (82).

We noted significant differences in body weight changes over the twenty-one day period between the ZIKV-IG and the Gamunex^®^ control treatment group (**Figure 4A**). Analysis of changes in the body weight demonstrated that there was a statistically significant difference between ZIKV-IG and Gamunex^®^ (p=0.03) and isotype control groups at study days 7 (p=0.04) and 14 (p=0.001) (**Figure 4A**). We did not observe any weight change differences between the 4G2 and isotype control mAb treated mice, as anticipated by our unpublished data. By day 21, all groups had gained weight relative to their starting weights, although the ZIKV-IG treated group continued to weigh less than the control groups. To quantify the delay in weight gain, we measured the number of days it took to get back to the original starting weight for each group. The ZIKV-IG treatment group required 3 additional days to regain weight with a mean time to original weight of 16.5 days while isotype took 13.5 days (p<0.03 ZIKV-IG vs Isotype) and Gamunex^®^ took 12.3 days (p<0.005 ZIKV-IG vs Gamunex^®^) (**Figure 4A**). However, we did not observe any striking differences in the clinical scores between the treatments, with the time of onset, disease severity, and resolution of clinical score appearing to be similar between the groups (**Figure 4B**). Disease onset as determined by clinical score began in all groups by days 6-7, with the peak of disease severity being on days 8-9. By day 15, all mice had returned to a clinical score of 0, and no mortality was observed for any of the treatment groups during the 21-day period of the study (**Figure 4B**).

### The Role of Sex Differences in ZIKV-IG Treatment After ZIKV Infection

**Figure 5:**
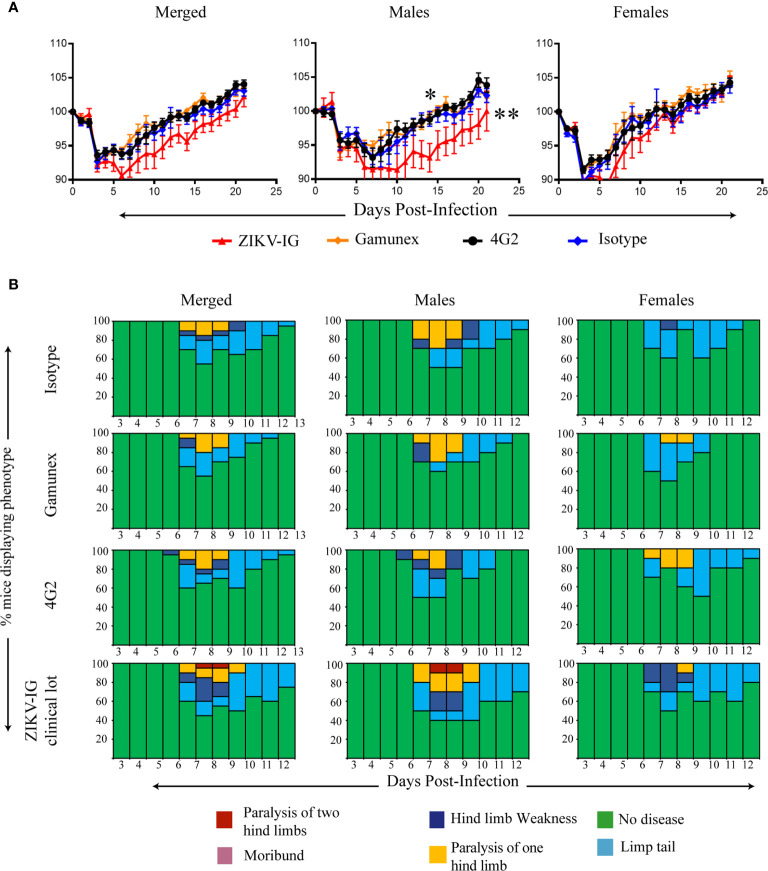
Role of sex differences on weight loss, and clinical score of ZIKV-infected *Ifnar1-/-* mice under ADE conditions. We separated the data from **Figure 4** based upon sex. Weight change **(A)** and clinical score **(B)** were monitored. The data reflects 10 male and 10 female mice per group. Mice were evaluated for signs of disease daily and graphed on each day as a percentage of mice displaying that disease indicator. Signs of disease range from no apparent disease, limp tail, hind limb weakness, hind limb paralysis, complete paralysis to death. Body weight comparison as single days was completed by Dunnett’s test, comparisons of weight over time was completed by 2-way ANOVA, while number of disease days was determined by Fisher exact test. Statistical differences are denoted by a star *p < 0.05, **p < 0.005.

To determine the impact of sex on ZIKV disease outcomes with IVIG administration, we separated the data collected for each treatment group by sex (n=10 males and n=10 females per group) and repeated our analysis (**Figure 5**). The male mice treated with the ZIKV-IG had significantly more severe weight loss between the ZIKV-IG treatment and controls (**Figure 5A**). Statistical analysis of the treatment groups showed that the ZIKV-IG treated male mice had significantly greater weight loss compared to male mice in other treatment groups starting on day 7 and through the completion of the study (p=0.001) (**Figure 5A**). Unlike the other treatment groups, the male ZIKV-IG treated *Ifnar1-/-* mice never fully regained weight back to baseline, indicating an enhancement of the duration of disease in ZIKV-IG treated male mice. With a similar comparative analysis for the female mice, we noted that like the male mice, the ZIKV-IG treated female *Ifnar1-/-* mice lost more weight during the course of the study compared to the control treated groups (**Figure 5A**). However, by the end of the study, unlike the male mice, the female mice treated with ZIKV-IG gained weight relative to their baseline weights and the weights similar to that of the control mice. Overall, the analysis of the female mice showed a milder disease course than was observed in the male mice infected with ZIKV (**Figure 5B**).

In summary, we completed these studies to determine if the ZIKV-IG would enhance ZIKV infection *in vivo* when delivered at a dose which represented the peak of *in vitro* based antibody enhancement and a strong level of antibody neutralization. Although we saw no differences in survival or clinical scores between the ZIKV-IG treated group and the control treated animals (**Figure 5B**), the analysis of mean body weight changes showed that treated animals lost significantly more weight compared to isotype controls, which suggests that the ZIKV-IG treatment increased disease severity. Upon evaluating the impact of sex on disease, the male mice developed a more severe disease upon ZIKV-IG treatment than female mice, as represented by overall increases in weight loss and a greater delay in return to original weight. This suggests that the ZIKV-IG administered at 0.050 mg/mouse enhanced disease and the impact of ZIKV-IG administration was most dramatically seen in the male *Ifnar1-/-* mice.

### Enhancement Potential of the ZIKV-IG *In Vivo* in *Ifnar1-/-* Mice Infected With DENV2

**Figure 6:**
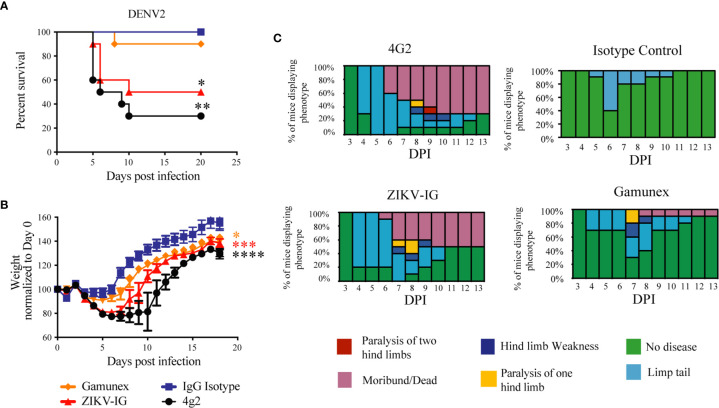
Weight loss, Survival and clinical score of DENV2 -infected *Ifnar* mice under ADE conditions. *Ifnar* ^-/-^ mice were transferred passively isotype control (0.020 mg of mouse anti-NP or 2 mg of Gamunex^®^) or enhancing mAbs (0.020 mg of anti-E (4G2) or 0.050 mg of ZIKV-IG) and then infected a day later with 10^4^ FFU of DENV-2 (strain D2S20) *via* an intravenous route. Survival **(A)** weight change **(B)** and clinical score **(C)** were monitored, and the data reflects 10 mice per group. Treatment with either ZIKV-IG or 4G2 increased the susceptibility of mice to severe DENV2 disease, significance in comparison to the isotype control mAb is provided in black (*) and significance in comparison to Gamunex^®^ is provided in red (*). Mice were evaluated for signs of disease daily and graphed on each day as a percentage of mice displaying that disease indicator. Signs of disease range from no apparent disease, limp tail, hind limb weakness, hind limb paralysis, complete paralysis to death. Mortality curve was analyzed by Log-rank test, Body weight comparison on single days was completed by Dunnett’s test, comparisons of weight over time was completed by 2-way ANOVA, while number of disease days was determined by Fisher exact test. Statistical differences are denoted by a star *p < 0.05, **p < 0.005, ***p < 0.0005, ****p < 0.0001.

To evaluate the ADE potential of a 0.050 mg/mouse dose of ZIKV-IG treatment on subsequent DENV2 infection, 40 *Ifnar1-/-* mice (n=10 per group, 5 males and 5 females) were treated with ZIKV-IG and control articles *via* IP route similar to the ZIKV ADE *in vivo* study, and 24 hours later were challenged *via* the IV route with 1 x 10^4^ FFU/mouse of the mouse adapted DENV2 strain, D2S20. Unlike the studies with ZIKV, the 4G2 treated *Ifnar1-/-* mice infected with DENV are considered the positive control for enhanced disease and the isotype as the negative control, as previous studies done by our group and others demonstrated the ability of 4G2 to induce ADE when administered one day prior to infection with DENV (38, 76). Weight loss, mortality, and clinical disease were monitored for 21 days following DENV challenge (**Figure 6**).

For the survival studies, unlike the studies with ZIKV, some of the antibody treated mice succumbed to DENV2 infection (**Figure 6A**). As anticipated, all the animals challenged with DENV2 and treated with isotype control survived to the end of the study. Seven of the ten (70%) animals treated with 4G2 succumbed to infection (p<0.001 versus isotype), and five of the ten animals (50%) treated with ZIKV-IG succumb to DENV2 before the end of the study (p<0.01 versus isotype). One of the ten Gamunex^®^, treated mice (10%) succumbed to DENV2 prior to the completion of the study. The mean time to death (MTD) for the 4G2 and ZIKV-IG groups were 7.5 and 10 days, respectively. Statistical analysis of the results revealed that there was no difference in survival between the ZIKV-IG treatment mice and the 4G2 treated positive controls in survival or MTD. The similarity between the ZIKV-IG and the 4G2 group in percent survival and MTD suggest that ZIKV-IG treatment is able to induce ADE in our animal model of DENV2 disease.

The analysis of the weight loss demonstrated that, independent of treatment, all animals lost the most weight between days 3 and 5 post-infection. This anticipated result is an indication of the establishment of infection in the susceptible *Ifnar1-/-* mice (**Figure 6B**). Analysis comparing the mean body weight change over time of the 4G2, Gamunex^®^, or ZIKV-IG treated groups demonstrated significant differences when compared to the isotype control group (**Figure 6B**). Importantly, ZIKV-IG treated animals lost as much weight over time as the 4G2 control group and significantly more weight compared to isotype control group between day 0 and day 5 (Isotype vs ZIKV-IG p=0.006; Gamunex^®^ vs ZIKV-IG p=0.044; 4G2 vs ZIKV-IG p=0.840).

Upon analysis of the clinical score, both ZIKV-IG and 4G2 increased the severity of DENV2 disease (day 0 vs day 5: isotype vs ZIKV-IG p=0.0116; isotype vs 4G2 p=0.0005) (**Figure 6C**). This indicates that the ZIKV-IG treatment had a similar effect on disease course as 4G2 and a negative impact on the course of disease relative to the isotype control. When considering the duration of disease, both ZIKV-IG and 4G2 treatment resulted in a longer duration of disease relative to both the isotype control and Gamunex^®^ control, with the average number of disease days being 6 vs. 12 when comparing isotype mAb to 4G2 and 7.5 and 9.5 days when comparing Gamunex^®^ to ZIKV-IG. Due to the limited number of animals used for each treatment group, we did not perform statistical analysis on the potential differences between the male and female mice in the individual treatment groups.

The primary objective of this study was to evaluate the effect of ZIKV-IG at a sub-neutralizing concentration of antibody on subsequent DENV infection due to the potential that sub-neutralizing concentrations of anti-flavivirus antibody could lead to ADE of infection, thereby increasing disease. As we had seen with ZIKV, pretreatment of mice with ZIKV-IG appeared to enhance DENV2 disease *in vivo*. Statistical analyses of both the survival and body weight demonstrated that both ZIKV-IG and 4G2 increased DENV2 disease in comparison to their respective controls and there were no statistical differences between treatment with 4G2 and ZIKV-IG, indicating that administration of a sub-neutralizing dose of ZIKV-IG to *Ifnar1-/-* mice one day prior to DENV infection resulted in an ADE-like disease as seen with pretreatment of 4G2. From these results, we can conclude that at a dose of 0.050 mg/mouse, ZIKV-IG contributes to an increase in DENV mortality and morbidity which is indicative of ADE.

### *In Vivo* Zika Virus Replication With Protective Versus Enhancing Doses of ZIKV-IG

**Figure 7:**
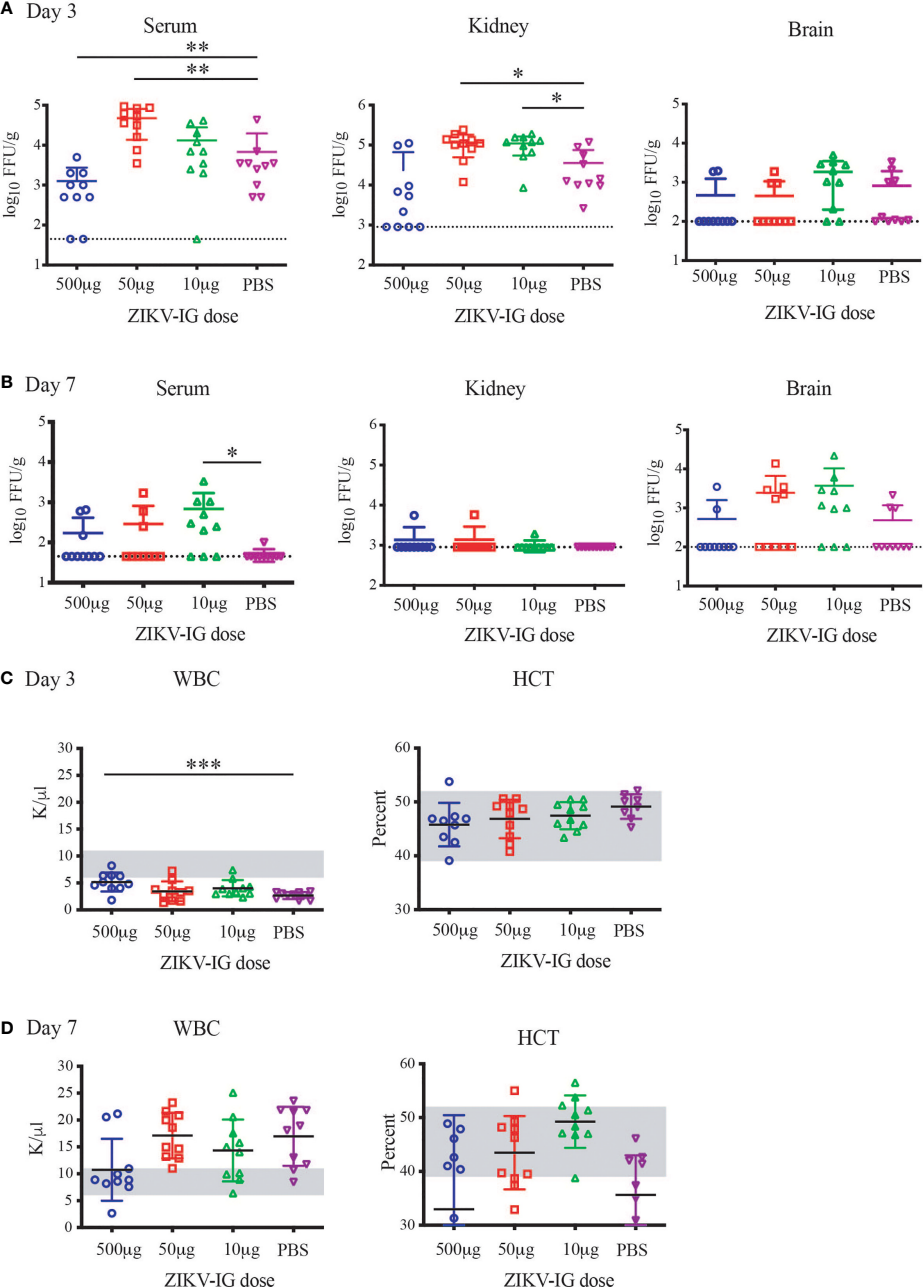
Alterations in Viral Replication and hematology from protective versus enhancing doses of ZIKV-IG. *Ifnar* ^-/-^ mice were infected with ZIKV (10^5^ FFU) *via* an intravenous route. *Ifnar* ^-/-^ mice were pre-treated with a protective concentration of ZIKV-IG (0.500 mg/mouse) or enhancing amounts of ZIKV-IG (0.050 and 0.010 mg of ZIKV-IG). Levels of virus were determined from samples harvested 3 days **(A)** or 7 days **(B)** after infection FFA. Data are shown as log 10 FFU/ml for serum or log 10 FFU/g for organs from n = 10 mice (5 male and 5 female) per condition. The dotted line represents the limit of sensitivity of the assay and error bars indicate SD. Asterisks indicate values that are statistically significant compared to PBS treated mice. White blood cell counts and hematocrit were evaluated at days 3 and 7 **(C, D)**. The grey region is the normal range of male and female C57BL/6 mice of the same age. Viral titer was evaluated by Mann Whitney *p < 0.05, **p < 0.005, ***p < 0.0005.

To understand the impact of ZIKV-IG treatment dose on viral replication and dissemination, we treated 8 to 10 week old mice with one of three doses of ZIKV-IG (0.500 mg, 0.050 mg, or 0.010 mg/mouse) or PBS *via* IP route. It has been previously demonstrated that a treatment dose of 0.500 mg/mouse of ZIKV-IG antibody protects *Ifnar1-/-* mice from ZIKV disease (52). Our results presented in **Figure 4** show that a dose of 0.050 mg/mouse can enhance ZIKV disease based upon weight loss and we anticipated a dose of 0.010 mg/mouse would not be of sufficient concentration to enhance disease. Twenty-four hours following antibody treatment, the mice were challenged *via* SC route with 1 x 10^5^ FFU/mouse of ZIKV strain PRVABC59. At day 3 (n=10 per group; 5 males and 5 females) and day 7 (n=10 per group; 5 males and 5 females) post-infection, mice were sacrificed for blood and tissue samples that were collected for viral burden analyses of both infectious virus (FFA, **Figures 7A, B**; **Figure S3**; **Table S1**) as well as white blood cell count and hematocrit (WBC and HCT, **Figures 7C, D**). Infectious virus levels as measured by FFA were significantly increased in serum viremia at day 3 in the 0.050 mg/mouse ZIKV-IG treatment group (p = 0.004) compared to PBS treated animals (**Figure 7A**). While infectious virus levels in the kidney were also significantly higher at day 3 in both the 0.050 mg/mouse (p = 0.022) and the 0.010 mg/mouse (p = 0.030) groups relative to the PBS control group (**Figure 7A**). The strongly neutralizing dose of ZIKV-IG (0.5 mg/mouse) resulted in a reduction of infectious virus in the serum, as would be predicted by previous work completed by Branche et al. (52). While we did observe viral replication within the liver, spleen, and brain, we did not observe significant differences between treatment groups (**Table S1** and **Figure S3**). The increase in viral titer within the serum and kidney strongly suggests that the increased disease observed (**Figure 4**) was due to an ADE of ZIKV infection.

Similar to day 3 post-infection, there was a significant increase in infectious serum viremia at day 7 in 0.010 mg/mouse ZIKV-IG treatment group (p = 0.015) compared to PBS treated animals (**Figure 7B**). In kidneys, ZIKV replication had ceased in most animals in all three treatment groups by day 7. While there was sustained viral replication within the liver and spleen, we did not observe any significant differences in viral titer between groups in those tissues (**Table S1**). Although there were no viral load differences in mean viral titer in the brain, there were more animals that still had virus replicating in the brain at the 0.050 mg (5/10 mice) and 0.010 mg (7/10) doses versus the 0.500 mg (2/10 mice) and PBS group (3/7 mice) (**Figure 7B**).

Severe viral infections, including arbovirus infections such as DENV, lead to alterations in white blood cell counts (WBC), and a correlate of severe DENV disease is represented by a drop in hematocrit (HCT). When evaluating the disease by WBC count, we observed a reduction in the WBC in all groups in comparison to the normal range for naive *Ifnar1-/-* mice (outlined in grey, **Figures 7C, D**). With the 0.500 mg/mouse ZIKV-IG protective dose the reduction in WBC is significantly less severe than the PBS group (p=0.0005) (**Figure 7C**). At the same time point, there were no changes in HCT levels outside of the normal range (**Figure 7C**), as would occur in a severe DENV infection. At seven days post infection, there was a return to the normal range of WBC in the 0.500 mg/mouse ZIKV-IG protective dose, while all other groups had an increase in WBC in response to infection. At day 7 most mice independent of group had a HCT within normal range (**Figure 7D**). The lack of alterations in hematocrit could be representative of either the low severity of disease or a potential characteristic of ZIKV infection.

To further understand the differences of sex on the disease that was observed during ZIKV infection (**Figure 5**), we separated the viral load based upon sex, where we observed a clear bias that could explain the increased disease within the male mice (**Table S1** and **Table 4**). We observed an increased viral load by FFA in male but not female animals within the serum and kidney at day 3 in the 0.050 mg/mouse ZIKV-IG group (p = 0.012 and p = 0.037), on day 7 in the 10 mg/mouse ZIKV-IG vs PBS (p = 0.044) (**Table S1**). There was no significant difference in infectious virus levels at day 3 in kidney for animals treated with 0.010 mg/mouse ZIKV-IG compared to PBS.

**Table 4:** Analysis of viral load in male and female mice on days 3 and 7.

Tissue	Treatment Group	Day 3			Day 7		
		Male	Female	Male + Female	Male	Female	Male + Female
Kidney	ZIKV-IG 0.50 mg/mouse	0.034*	0.403	0.092	0.424	1.000	1.000
	ZIKV-IG 0.05 mg/mouse	0.037*	0.095	0.022*	0.424	1.000	1.000
	ZIKV-IG 0.01 mg/mouse	0.095	0.060	0.030*	0.424	1.000	1.000
Serum	ZIKV-IG 0.50 mg/mouse	0.456	0.011*	0.106	1.000	0.180	0.703
	ZIKV-IG 0.05 mg/mouse	0.012*	0.205	0.004*	0.441	0.424	0.703
	ZIKV-IG 0.01 mg/mouse	0.141	0.672	0.715	0.044*	0.072	0.015*

*Significant Wilcoxon rank sum p-value (<= 0.05).

The primary goal of this study was to understand the quantitative relationship between antibody neutralization and enhancement for the human polyclonal antibody response and the impact of that relationship on antibody-dependent disease development. A secondary goal was to define the dose range that could potentially exacerbate disease by ADE by quantifying viral load. In this study, a dose of 0.05 mg/mouse of ZIKV-IG increased disease severity for ZIKV and DENV2 infection. Upon investigation, both the 0.050 mg/mouse and 0.010 mg/mouse increased viral load over PBS treated controls, providing a potential mechanism of increased disease severity during *in vivo* ZIKV infection.

## Discussion

ZIKV has become endemic within mosquito populations and non-human primates throughout Asia, Central and South America (83). Although the number of ZIKV in infections throughout the world is low, it is likely that similar to the cyclic endemicity of DENV, ZIKV is in an interepidemic period with high potential to cause future epidemics. Preparation for future ZIKV epidemics requires the continued development of new ZIKV therapeutics. Here we investigated the safety profile of a ZIKV human hyperimmunoglobulin, called ZIKV-IG known to be protective against severe ZIKV disease in a mouse model of infection (52).

The potential for the ADE of flavivirus infection must be considered when investigating the use of ZIKV antibody therapeutics. ADE occurs when virus-specific antibodies bind to the virus at a stoichiometry that is insufficient for neutralization, which then allows infection of cells *via* the Fc receptor causing an increase in the total number of infected cells. To begin to define the boundary of therapeutic efficacy and potential for ADE, we investigated the ability of ZIKV-IG to enhance ZIKV and DENV infection *in vitro*. The ability of mAbs and human polyclonal serum to drive ADE of ZIKV and DENV infection *in vitro* are well documented (40, 45, 47, 66, 68, 76). However, the characteristics of purified human hyperimmunoglobulin to neutralize ZIKV and DENV as well as cause ADE in both homologous and heterologous infections has not been well characterized. Here we used the most common assays, a Vero based FRNT assay to measure antibody neutralization and a K562 based Fc-mediated antibody based enhancement assay to measure the neutralization and enhancement profile of ZIKV-IG. ZIKV-IG was capable of both neutralizing and enhancing activity for ZIKV as well as multiple DENV serotypes (**Figures 1**, **2**), observations that are similar to serum derived from individuals who had multiple flavivirus infections (33, 49, 50, 84).

In order to use the *in vitro* data to predict the potential for severe disease from ZIKV-IG we overlayed the neutralization and ADE profile to begin to predict the threshold for protection versus ADE. mAbs generated against WNV, and DENV have demonstrated that ADE is possible when an insufficient number of antibody epitopes are occupied to cause neutralization (73, 75), this occurs when mAbs are at their FRNT_50_ concentration or lower. When we evaluated ZIKV-IG, the peak of ADE occurred close to the FRNT_90_ value of ZIKV-IG, indicating a potential difference in the stoichiometry between mAbs and polyclonal sera for antibody neutralization versus enhancement. Our experiments using HMDMs show that a broad range of ZIKV-IG concentrations can drive ADE in primary cells. Overall, the *in vitro* ADE results highlight that increased infection can occur when the concentration of neutralizing antibodies becomes insufficient to completely neutralize virus.

The ZIKV-IG therapeutic we have investigated was strongly neutralizing *in vitro*, although here we demonstrated that at lower concentrations was able to enhance ZIKV and DENV infection *in vitro*. While donors were selected for ZIKV specificity, we determined that the ZIKV-IG contained cross-reactive neutralizing and enhancing antibody to DENV1-4, commonly associated with antibody responses to multiple flavivirus infections (50, 84, 85). Human derived anti-DENV and ZIKV antibodies generated by natural infection or vaccination have led to mixed results in animal models of infection, with some experimental systems demonstrating ADE and other experimental systems demonstrating protection in the absence of ADE [Reviewed in (68, 86)].

With recent epidemiological evidence demonstrating that prior ZIKV infection can lead to increased human DENV disease (33), it was important to investigate the potential for ZIKV-IG to cause ADE *in vivo*. At a dose of 0.050 mg/mouse ZIKV-IG was able to enhance both ZIKV and DENV disease of *Ifnar1-/-* (**Figures 5**, **6**), as demonstrated by increased weight loss and clinical score over Gamunex^®^, a control IVIG. To understand the mechanism of enhanced disease we investigated the viral load in mice given ZIKV-IG. In these experiments, we observed increased viral titers at two different doses of ZIKV-IG, which supports the potential for polyclonal therapeutics like ZIKV-IG to cause ADE when the concentration drops below a value that drives neutralization. This is consistent with reports that polyclonal anti-DENV antibody responses are capable of enhancing ZIKV disease within STAT2-/- mice (54, 87) as well as that anti-ZIKV immune plasma can enhance ZIKV infection in *ifnar1* -/- mice (68). This demonstrates the ability of hyperimmunoglobulin to have the potential for enhancement of both homologous (ZIKV) and heterologous ADE. However, we acknowledge that our study and most studies utilize innate immunodeficient mice, IFNAR1-/-, where type I interferon signaling may influence the balance between protection and ADE.

An interesting phenomenon we observed in this study is the role of sex differences in ZIKV-IG treatment outcomes, which to our knowledge, was not investigated in other cases of ADE of ZIKV infection. In the evaluation of viral loads stratified by sex there were significant increases in live virus levels in male, but not female animals treated with ZIKV-IG (**Figure 5**). This type of sex effect has not been previously observed and this could be due to the large number of animals used in the current study compared to previous studies. Although the underlying mechanism for a sex effect associated with ADE of ZIKV or DENV is not known. In epidemiological studies of DENV and ZIKV infection, the probability of symptomatic infection was equal in both male and females (88).

This study has investigated the role ZIKV-IG, a polyclonal antibody based ZIKV therapeutic could play in antibody mediated protection and ADE. Here we have defined the critical antibody concentration that leads to a reduction in viral load and which could lead to ADE of either ZIKV or DENV infection. Overall, ZIKV-IG hyperimmunoglobulin at high concentrations was capable of reducing viral load, but at lower concentrations was capable of driving enhanced ZIKV and DENV2 disease and that the disease potential occurs when the concentration of ZIKV-IG is at a sub-neutralizing dose *in vivo* and the peak enhancement dose *in vitro*.

## Data Availability Statement

The original contributions presented in the study are included in the article/**Supplementary Material**. Further inquiries can be directed to the corresponding authors.

## Ethics Statement

The animal study was reviewed and approved by Saint Louis University IACUC.

## Author Contributions

MH, SS, AP, and JB were responsible for the conceptualization of the study. TS, MH, EB, KM, and JB performed the experiments and analyzed the data. SS, AP, and JB provided the resources. TS, EG, MH, and JB writing—original draft preparation. MH, EG, BG, ES, EB, SS, SR, SK, AP, and JB, writing—review and editing of the final draft. AP and JB were responsible for funding acquisition. All authors contributed to the article and approved the submitted version.

## Funding

This research was funded by Saint Louis University startup funding and a research contract from Emergent BioSolutions to SK, SS, AP and JB. The funder was not involved in the study design, collection, analysis, interpretation of data, the writing of this article or the decision to submit it for publication. This work was also supported by National Institutes of Health grant F31 AI152460-01 from the National Institute of Allergy and Infectious Diseases (NIAID) awarded to MH, NIH grant R0112781495 from the NIAID awarded to AP and JB.

## Conflict of Interest

XH, DB, TC, CN, DT, and SK are employees of Emergent BioSolutions.

The remaining authors declare that the research was conducted in the absence of any commercial or financial relationships that could be construed as a potential conflict of interest

## Publisher’s Note

All claims expressed in this article are solely those of the authors and do not necessarily represent those of their affiliated organizations, or those of the publisher, the editors and the reviewers. Any product that may be evaluated in this article, or claim that may be made by its manufacturer, is not guaranteed or endorsed by the publisher.
