# Supplementary material for: The Ability of Zika virus Intravenous Immunoglobulin to Protect From or Enhance Zika Virus Disease
Source: Front Immunol. 2021 Sep 6;12:717425. doi: 10.3389/fimmu.2021.717425 (PMC8450494; doi:10.3389/fimmu.2021.717425)
Supplement: Supplementary file 1 [file DataSheet_1.pdf]

Supplemental Fig 1. Monoclonal Antibody Mediated Enhancement of Zika and Dengue virus

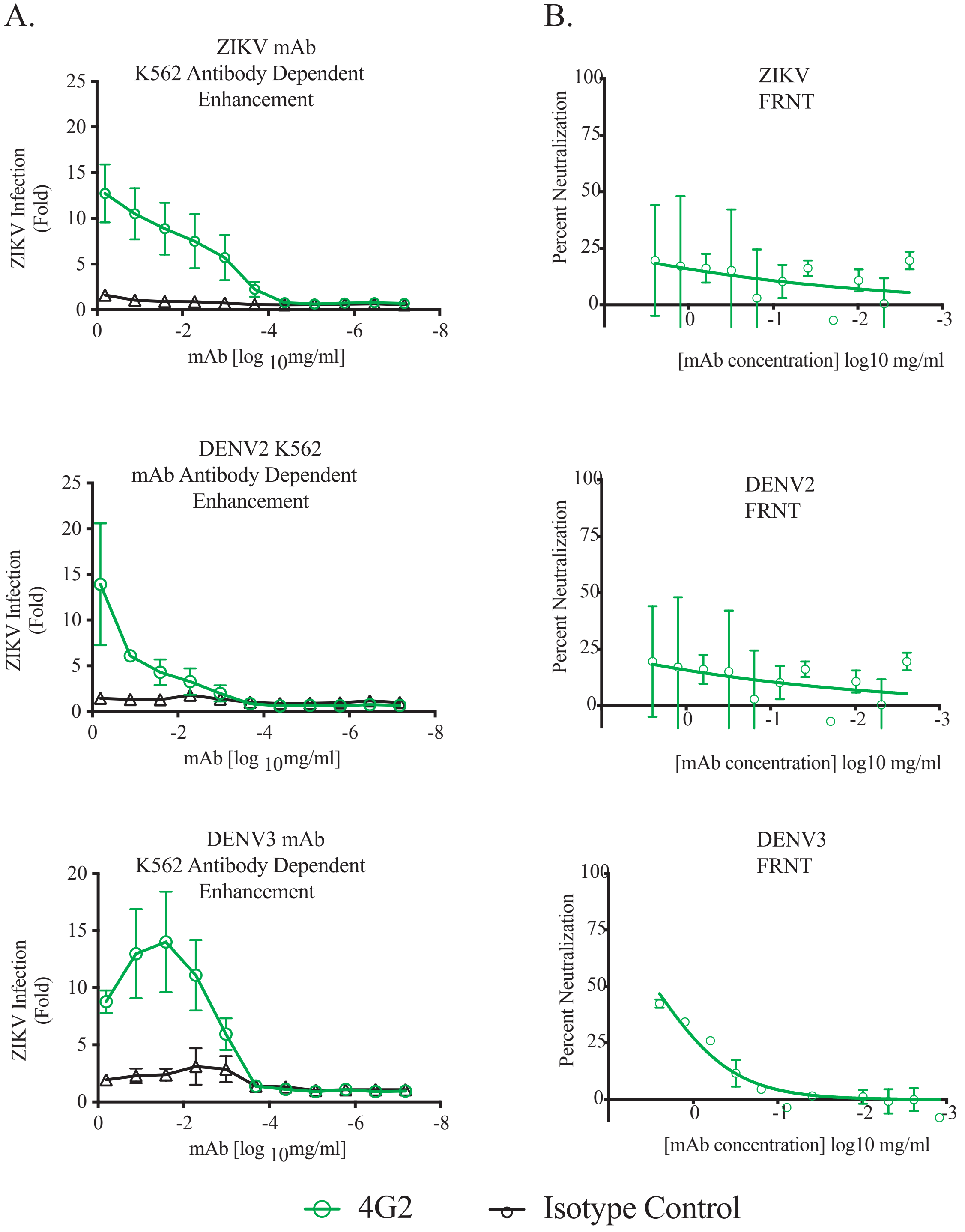

Supplemental Figure. 1 A. Antibody dependent enhancement activity: Infection of K562 cells with A. ZIKV (strain PRVABC59), B. DENV2 (strain D2S20), C. DENV3 (strain C0360) in the presence of monoclonal antibody. In the presence of 4G2 there was increased infection of K562 cells, ADE, that was not seen in the isotype control. All graphs shown are a compilation of 3-5 independent experiments. The data is presented as the ratio of viral infection in the presence of antibody to viral infection in the absence of antibody. **B.** Antibody mediated neutralization of ZIKV, DENV2 and DENV3 measured by focus reduction neutralization tests on Vero cells.

Supplemental Figure 2

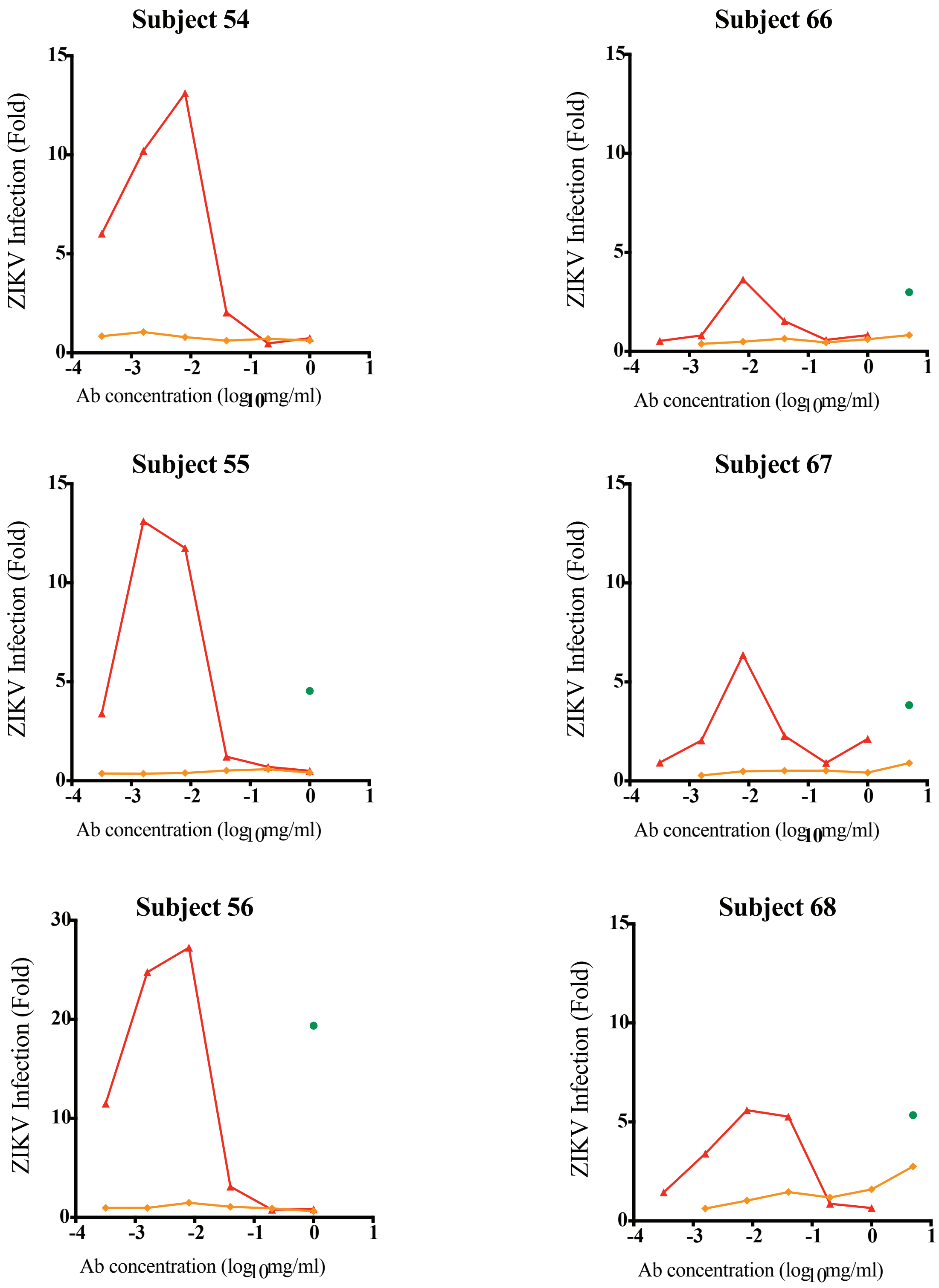

Supplemental Fig 2. ADE in primary human monocyte derived macrophages. ZIKV (strain FSS13025) was pre-incubated at a multiplicity of infection (MOI) of 1 for 30 min with serial dilutions of either ZIKV-Ig (in red) or Gamunex (in orange). After incubation, the mixtures were added to primary human monocyte derived macrophages. Following 24 hours of infection, cells were harvested and stained with fluorescein-labeled pan-flavivirus specific monoclonal antibody (4G2) and analyzed by flow cytometry to determine the percentage of infected cells. The percentage of human macrophages infected with ZIKV only was used as a baseline of infection to calculate the ADE. Data shown as single points that represent the mean of one experiment completed in duplicate from three individual donors.

Supplemental Figure 3

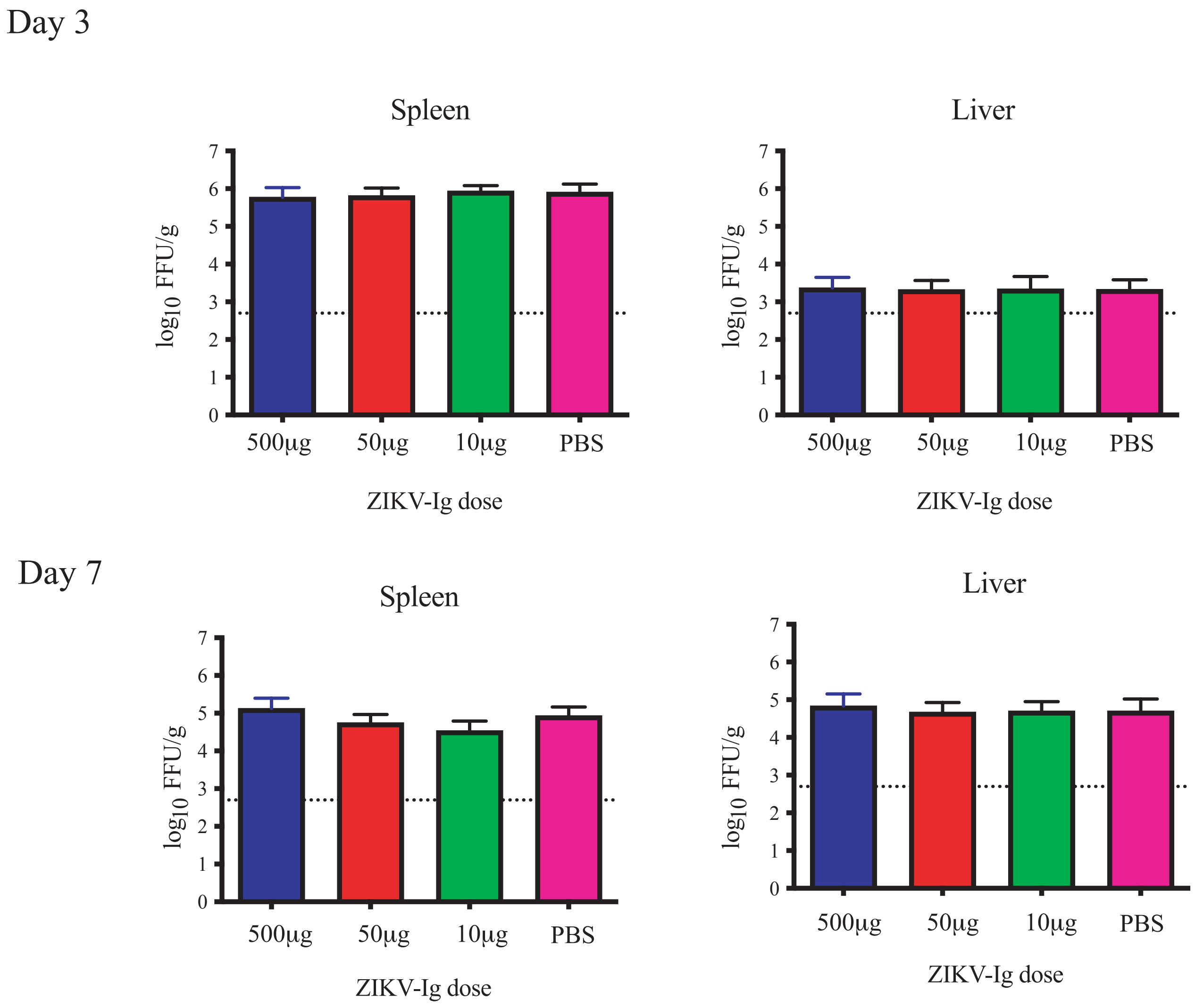

Supplemental Figure 3. Viral Replication in Ifnar-/- mice treated with ZIKV-Ig. Ifnar-/- mice were pre-treated with a protective concentration of ZIKV-Ig (0.500 mg/mouse) or enhancing amounts of ZIKV-Ig (0.050 and 0.010 mg of ZIKV-Ig) then infected with ZIKV (10^5 FFU) via an intravenous route.. Levels of virus were determined from samples harvested 3 days (**A**) or 7 days (**B**) after infection FFA. Data are shown as log 10 FFU/g for organs from n = 10 mice (5 male and 5 female) per condition. The error bars indicate SD. Asterisks indicate values that are statistically significant compared to PBS treated mice. (**, P < 0.01, ***, P < 0.001, ****, P < 0.0001; Mann-Whitney test).

Supplemental Table 1

		Day 3<br>Wilcoxon rank sum p-value vs. placebo		Day 7<br>Wilcoxon rank sum p-value vs. placebo	
Tissue	Treatment Group	Male	Female	Male	Female
Brain	ZIKV-IG 0.50 mg/mouse	0.025*	0.824	0.424	1.000
	ZIKV-IG 0.05 mg/mouse	0.331	0.347	0.045*	0.797
	ZIKV-IG 0.01 mg/mouse	0.600	0.287	0.119	0.265
Kidney	ZIKV-IG 0.50 mg/mouse	0.034*	0.403	0.424	1.000
	ZIKV-IG 0.05 mg/mouse	0.037*	0.095	0.424	1.000
	ZIKV-IG 0.01 mg/mouse	0.095	0.060	0.424	1.000
Liver	ZIKV-IG 0.50 mg/mouse	0.209	0.451	1.000	0.210
	ZIKV-IG 0.05 mg/mouse	0.398	0.236	0.296	0.144
	ZIKV-IG 0.01 mg/mouse	0.833	0.332	1.000	0.753
Serum	ZIKV-IG 0.50 mg/mouse	0.456	0.011*	1.000	0.180
	ZIKV-IG 0.05 mg/mouse	0.012*	0.205	0.441	0.424
	ZIKV-IG 0.01 mg/mouse	0.141	0.672	0.044*	0.072
Spleen	ZIKV-IG 0.50 mg/mouse	0.142	1.000	0.037*	0.403
	ZIKV-IG 0.05 mg/mouse	0.209	1.000	0.835	0.060
	ZIKV-IG 0.01 mg/mouse	0.402	0.210	0.600	0.037*

* = Significant Wilcoxon rank sum p-value<= 0.05 (unadjusted).

Supplemental Table 1. Analysis of viral titer by focus forming assay in male and female mice for days 3 and 7.
